# Anti-algal activity of the 12-5-12 gemini surfactant results from its impact on the photosynthetic apparatus

**DOI:** 10.1038/s41598-021-82165-9

**Published:** 2021-01-27

**Authors:** Konrad Krajewski, Katarzyna Łudzik, Aneta Żabka, Justyna Teresa Polit, Anna Zawisza, Janusz Maszewski

**Affiliations:** 1 Department of Cytophysiology, Faculty of Biology and Environmental Protection, University of Lodz, ul. Pomorska 141/143, 90-236 Lódź, Poland; 2 Department of Physical Chemistry, Faculty of Chemistry, University of Lodz, ul. Pomorska 163/165, 90-236 Łódź, Poland; 3 Frank Laboratory of Neutron Physics, Joint Institute for Nuclear Research, Dubna, Russia; 4 Department of Organic and Applied Chemistry, Faculty of Chemistry, University of Lodz, ul. Tamka 12, 91-403 Łódź, Poland

**Keywords:** Conservation biology, Freshwater ecology, Ecology, Secondary metabolism

## Abstract

A rapid amplification of algal population has a negative impact on the environment and the global economy. Thus, control of algal proliferation is an important issue and effective procedures which reduce algal blooms and control algal fouling are highly desired. Gemini surfactants are considered to have a low environmental impact, therefore they seem to be a promising group of detergents which could reduce algal blooms in water systems. Furthermore, due to their emulsifying properties they could replace algaecides added to antifouling paints and decrease algae adhesion to various surfaces. In this study the toxic effect of the 12-5-12 gemini surfactant was investigated on Chlorella cells and close attention was paid to a potential mechanism of its action. At the high cell density (10.05 × 10^7^ cells/mL) a dose-dependent cell death was found and the IC_50_ value was reached at the concentration of 19.6 µmol/L after 72-h exposure to the surfactant. The decrease in chlorophyll autofluorescence shows that the photosynthetic apparatus seems to be the target of the tested compound. The presented studies indicate that gemini surfactants could effectively reduce algal blooms in water systems, and if added to paints, they could decrease algal growth on external building walls or other water immersed surfaces.

## Introduction

Climate change and water eutrophication lead to a rapid and uncontrolled growth of an algal population^1,2^. Algal blooms appear mainly during summer and they may negatively affect the environment, the economy and even cultural ecosystem services^3–5^. A rapid increase in the algal population decreases the amount of oxygen and limits light availability leading to death of animals and plants, thus reducing the biodiversity of aquatic ecosystems^3,6,7^. The algal blooms cause significant losses in fish farming^8–11^ and may have negative impact on agriculture. Harmful algal bloom (HAB) occurring in water reservoirs may deteriorate water quality, making it unsuitable for irrigation and consumption^10,12–14^. Some algal species show adhesive properties to different surfaces and may induce damages to water irrigation systems, cooling systems, drinking water distribution systems and other materials immersed in water^15–18^. Furthermore, algae were also found to grow on external building walls causing biodegradation and damage of technical materials^19,20^. Since rapid algae proliferation and biofilm formation impact negatively the economy, there is an ongoing battle against uncontrolled algal growth which concerns two approaches: reduction of algal blooms in water systems and prevention of algae adhesion to various surfaces. Algaecides may show high toxicity to aquatic environment^21,22^, thus researches are still looking for new compounds with a lower environmental impact. Some studies concentrate on new antifouling compounds and methods^23–26^ while others point to bacteria cultures that cause death of algal cells^27–29^ or focus on peptides and biosurfactants with an algaecidal activity^30,31^. Various synthetic surfactants were also tested and cationic ones were found to have the strongest adverse effect on algal biomass^32,33^. However, monomer detergents were found to have a higher negative impact on the aquatic environment than dicationic ones^22^. Thus, gemini surfactants seem to be a promising group of synthetic detergents which could combine antialgal activity and antifouling properties. They might reduce algal blooms in water systems with a low impact on the environment, and due to emulsifying properties, they could substitute algaecides in antifouling paints, and thus reduce algal growth on water immersed surfaces and building facades. The cytotoxic effects and antibacterial activity of various gemini surfactants were studied intensively^34–37^, while their algaecidal properties are poorly described^22,38^.

**Figure 1:**
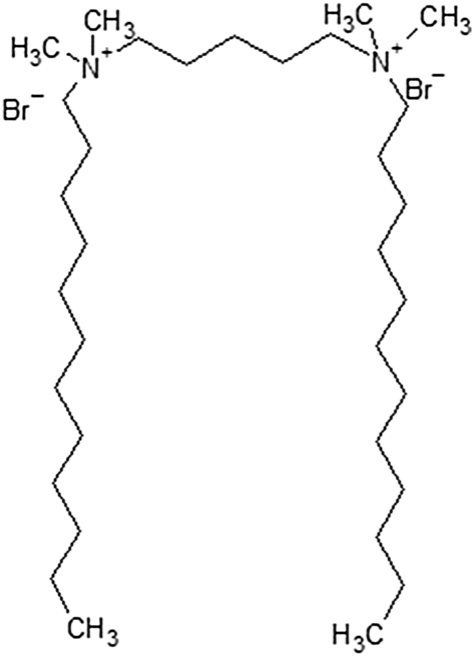
Chemical structure of pentylene-1,5-bis(dimethyldodecylammonium bromide).

The aim of this work was to investigate the algaecidal activity of pentylene-1,5-bis(dimethyldodecylammonium bromide), a dicationic surfactant described by formula 12-5-12 where 12 indicates the number of carbon atoms in each of alkyl tails and 5 indicates the number of carbon atoms in the polymethylene spacer (Fig. 1). Gemini surfactants with 12 carbon atoms are one of the most frequently studied dicationic molecules. Their physico-chemical properties and biological activity tested on animal and bacterial cells are widely discussed, therefore many comparative data are available. Some studies indicate that gemini surfactants with 12 carbon atoms in alkyl tails show higher negative impact on cell viability compared to their longer-tailed counterparts^35,39^. Interestingly, long alkyl chains allow for higher conformational freedom which result in maintenance the high order of a lipid bilayer^40^. However toxic effects of an individual compound seem to depend also on a cell type, and the long-tailed molecules may significantly reduce cell viability in some cases^35,37^. Nevertheless, gemini surfactants with 12 carbon atoms in alkyl chain are less hydrophobic and show higher value of the critical micelle concentration (CMC), compared to counterparts with longer alkyl tails^41^. Thus, the risk of micelles formation at the higher compound concentrations is significantly reduced. Although, among surfactant consisting of 12 carbon atoms in the alkyl tail, positive correlation between the length of the polymethylene spacer and the toxicity was found^35,37^, gemini surfactants with longer spacers are more hydrophobic^42^ and due to bending of the spacer, they show changes in the molecule conformation, which may result in the higher negative impact on lipid bilayer organization^35,40^. Thus, the balance between strong biocidal activity of the tested compound and possibly lesser impact on membrane disorganization, was the basis for the selection of surfactant containing the 5 carbon atoms spacer, which is long but still rigid enough to limit conformational changes in the surfactant structure. The mechanism of algaecidal activity of gemini surfactants has not been investigated in detail, therefore close attention is paid to cell viability, chlorophyll autofluorescence, plasma membrane potential and the production of reactive oxygen species (ROS). The toxicity of the 12-5-12 compound was tested on high cell density of *Chlorella vulgaris* which is one of the microalgas negatively affecting the environment and the economy. Apart from water blooming, Chlorella was found to form biofilm on different surfaces^43^ and take part in metal corrosion^44^. It may also foul industrial cooling systems^17^ and building facades^45^. The high cell density was used in our study to determine toxic properties of the tested compound at such cell concentrations which may appear near the surface of a scum formed during massive algal blooms^46^. Furthermore, the high cell density was found to correlate positively with increased number of cells attached to various surfaces^43^, and biofilm formation was previously studied at the cell concentrations reaching 10^7^ cells/mL^47^.

The obtained data indicate a strong algaecidal activity of the 12-5-12 surfactant and show that photosynthetic apparatus seems to be the target of the tested compound. Thus, our study not only indicates toxic effect of the 12-5-12 compound on algal cells but it may also trigger a discussion concerning a potential mechanism of its action.

## Results and discussion

### The 12-5-12 gemini surfactant exhibits strong algaecidal activity

**Figure 2:**
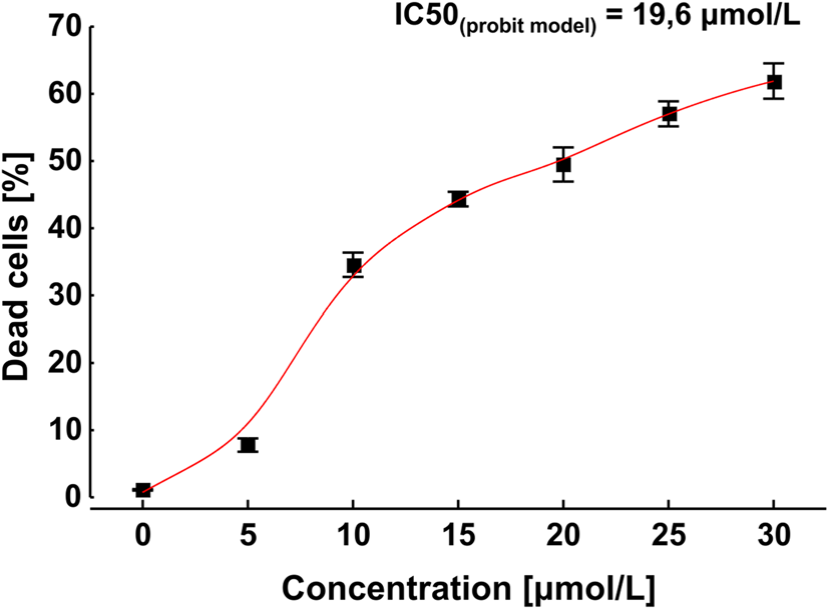
The percentage of dead cells after 72-h exposure to various concentrations of the 12-5-12 surfactant. An initial cell concentration at the level of 10.05 × 10^7^ cells/mL. The data points show a mean value and a standard deviation from three independent treatments. IC50 value was estimated with the probit model.

**Figure 3:**
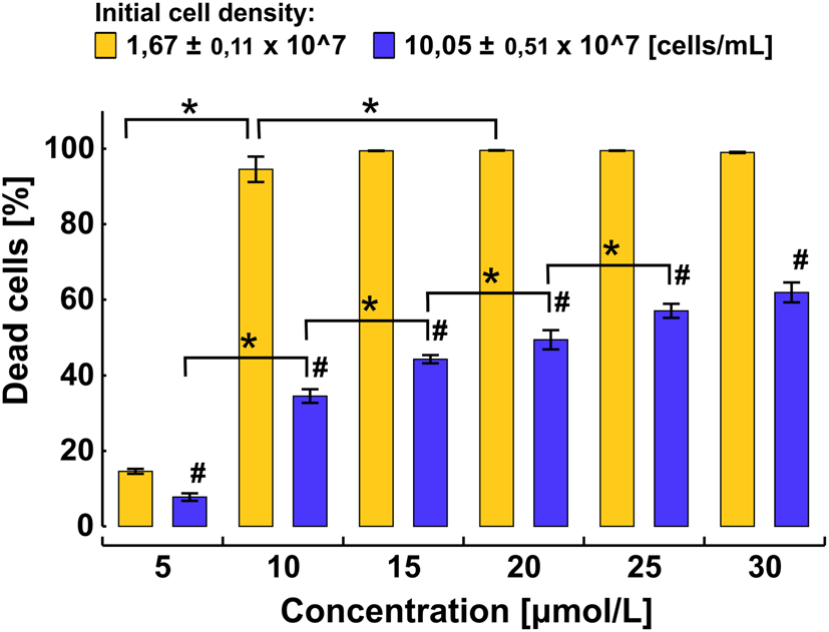
The comparison of a density-dependent effects on cell viability of 72-h exposure to various concentrations of the 12-5-12 surfactant. The columns show mean values and the error bars represent a standard deviation from three independent treatments. Statistical significance was assessed with the two-way ANOVA followed by the post-hoc Tukey's honestly significant difference (HSD) test. An asterisk indicates statistically significant differences between the compound concentrations and a number sign points to statistically significant differences between the various cell densities (p < 0.05).

The flow cytometric analysis indicated a dose-dependent cell death after 72-h exposure to the 12-5-12 surfactant and the IC_50_ value was reached at the concentration of 19.6 µmol/L (Fig. 2). The algaecidal effect depended strongly on an initial cell concentration. At the low cell density (1.67 × 10^7^ cells/mL) the level of dead cells reached 94.6% after exposure to the concentration of 10 µmol/L, and hardly any viable cells were observed after exposure to the higher concentrations. In turn, at the higher cell density (10.05 × 10^7^ cells/mL) the percentage of non-viable cells was lower and reached 34.6% at the concentration of 10 µmol/L. However, a successive increase in the fraction of dead cells was observed with rising compound concentrations (Fig. 3).

Cationic surfactants were found to show higher toxicity in comparison to anionic ones^48^, which may result from a negative charge being present on the surface of algal cells. However, among monomeric cationic detergents, their toxicity seems to depend on the alkyl chain length. Hexadecyltrimethylammonium bromide (CTAB) and hexadecyltrimethyl-ammonium chloride (CTAC), whose alkyl tails consist of 16 carbon atoms, show stronger algaecidal activity, in comparison to dodecyltrimethylammonium bromide (DTAB) and dodecyltrimethylammonium chloride (DTAC), whose alkyl tails consist of 12 carbon atoms only^49^. Liang and coworkers indicated the strong toxic effect of CTAB on *C. vulgaris* cells, and 50% reduction in cell viability was shown at the concentration of 1.64 µmol/L^50^. Similar effect was obtained for *Nannochloropsis gaditana* cells, and the IC_50_ value was reached at the concentration of 2.14 µmol/L. In the same study, IC_50_ value for DTAC was reached at higher compound concentrations (34.86 µmol/L)^22^. Although in our studies IC_50_ value for the 12-5-12 surfactant was reached at the concentration of approximately 20 µmol/L, initial cell density was 100–1000 times greater than that used in the studies described above. Thus, the 12-5-15 surfactant seems to show much higher algaecidal activity in comparison to DTAC and at least similar or higher, in comparison to CTAB. Thus, the obtained data indicate algaecidal activity of the 12-5-12 gemini surfactant on Chlorella cells, however the compound toxicity strongly depended on its concentration and the initial cell density. All further experiments were performed with the cell density ranging from 7.5 × 10^7^ to 10 × 10^7^ cells/mL.

### The 12-5-12 surfactant does not seem to damage plasma membrane directly

**Figure 4:**
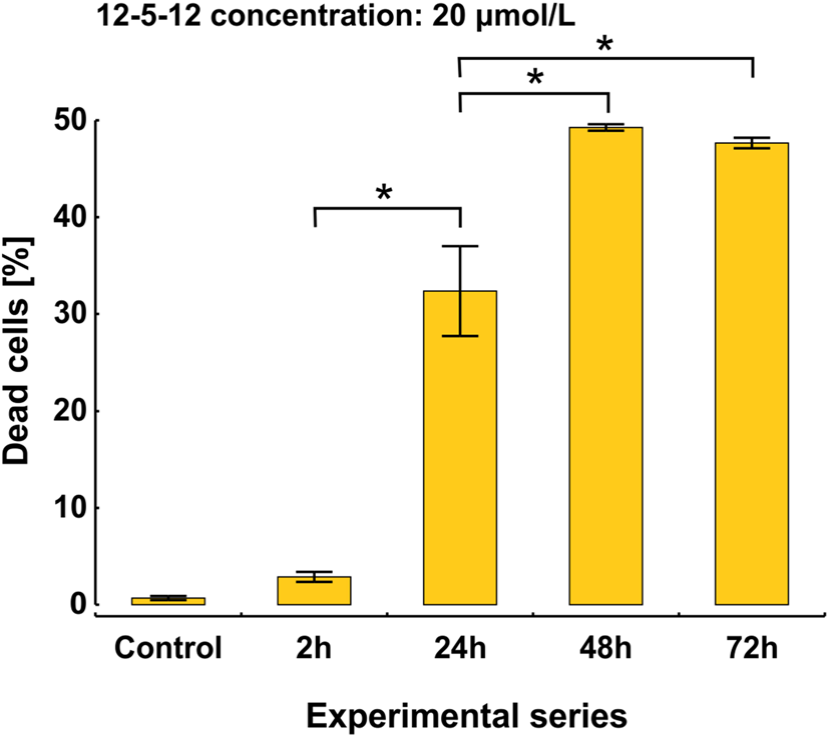
The time-dependent effects on the percentage of dead cells after exposure to the 12-5-12 surfactant at the concentration of 20 μmol/L. The columns show mean values and the error bars represent standard deviation from three independent treatments. Statistical significance was assessed with the one-way ANOVA followed by the post-hoc Tukey's honestly significant difference (HSD) test. An asterisk indicates statistically significant differences (p < 0.001).

It is accepted that surfactants destabilize plasma membrane integrity thus inducing cell death. To estimate dynamics of the 12-5-12 activity, algaecidal effects were studied after exposing Chlorella cells to its concentration of 20 µmol/L at different time points. After 2-h treatment the fraction of dead cells enlarged slightly up to 2.9%, however in comparison to the control no statistical significance was found. The number of non-viable cells increased with the prolonged incubation time to 32.4%, 49.3% and 47.7% after 24-h, 48-h and 72-h exposure, respectively (Fig. 4). The high percentage of viable cells after 2-h treatment might indicate that the 12-5-12 surfactant does not destabilize plasma membrane directly and toxic effects may result from the secondary damage of plasma membrane. To validate this hypothesis the plasma membrane potential was monitored with DiOC6(3) according to the previously described procedure^51^.

**Figure 5:**
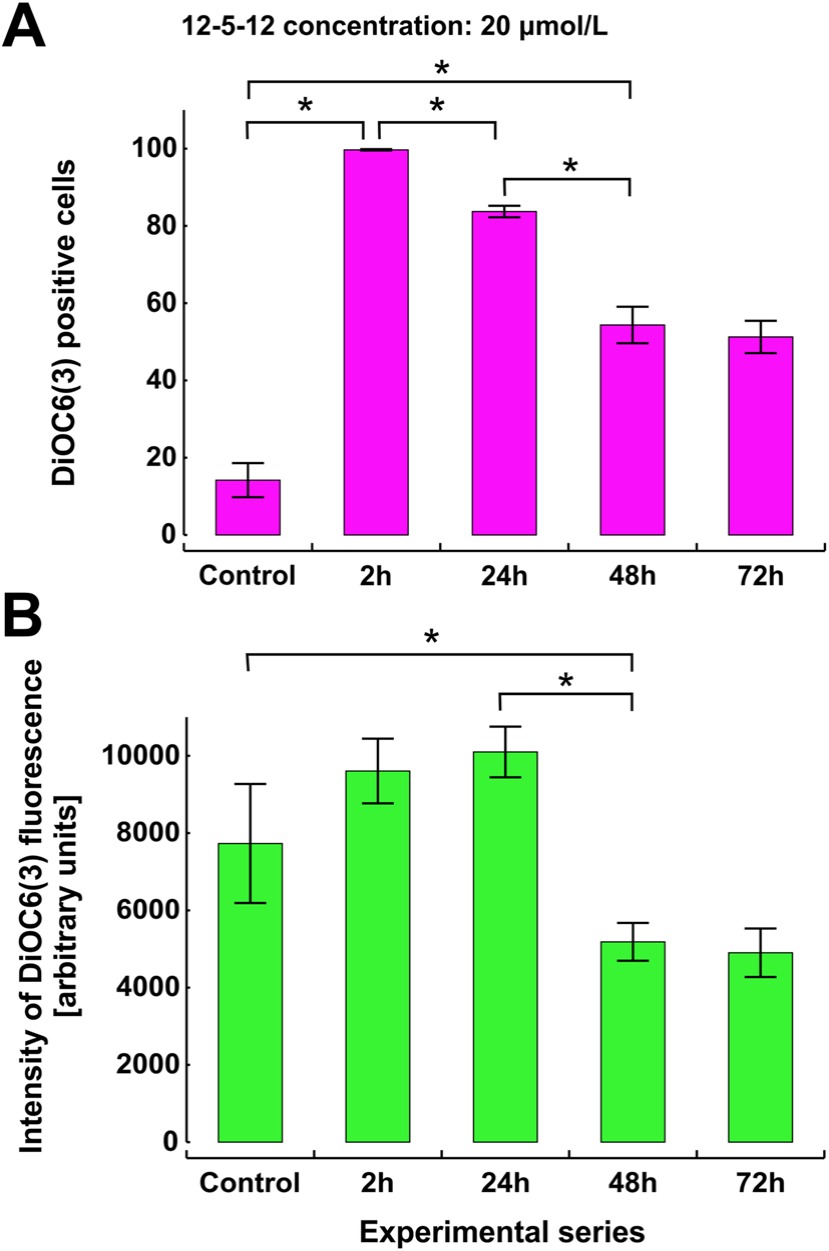
The changes in the membrane potential in the fraction of viable cells. The membrane potential was estimated with DiOC6(3) during the following hours of exposure to the 12-5-12 surfactant at the concentration of 20 μmol/L. (**A**) The percentage of DiOC6(3) positive cells. (**B**) The intensity of DiOC6(3) fluorescence. The columns show mean values and the error bars represent standard deviation from three independent treatments. Statistical significance was assessed with the one-way ANOVA followed by the post-hoc Tukey's honestly significant difference (HSD) test (p < 0.005 and p < 0.05 for (**A**) and (**B**) respectively).

After 2-h exposure to the 12-5-12 surfactant most of the living cells (99.7%) exhibited green fluorescence which indicates plasma membrane hyperpolarization. However, during following hours of incubation the number of DiOC6(3) positive cells decreased in the fraction of viable cells to the level of 51.3% after 72-h exposure (Fig. 5A). At the same time a reduction of fluorescence intensity below the level of control cells was also observed (Fig. 5B). The low number of dead cells after 2-h exposure and reversible changes in the membrane potential during the following hours of treatment may indicate that the integrity of plasma membrane is not rapidly lost upon the 12-5-12 application. For example, lipoplexes of the zwitterionic phospholipid 1,2-dimyristoyl-sn-glycero-3-phosphatidylcholine (DMPC) and a gemini surfactants were found to have little effect on plasma membrane^52^. Furthermore, the extent of plasma membrane disorganization was significantly reduced during a treatment with gemini surfactants consisting of shorter spacers and resembled the result obtained for DTAB^35^. Thus, algaecidal activity of the tested compound may result from changes in overall cellular metabolism which then affects the stability of lipid bilayer.

### The 12-5-12 surfactant reduces chlorophyll autofluorescence

**Figure 6:**
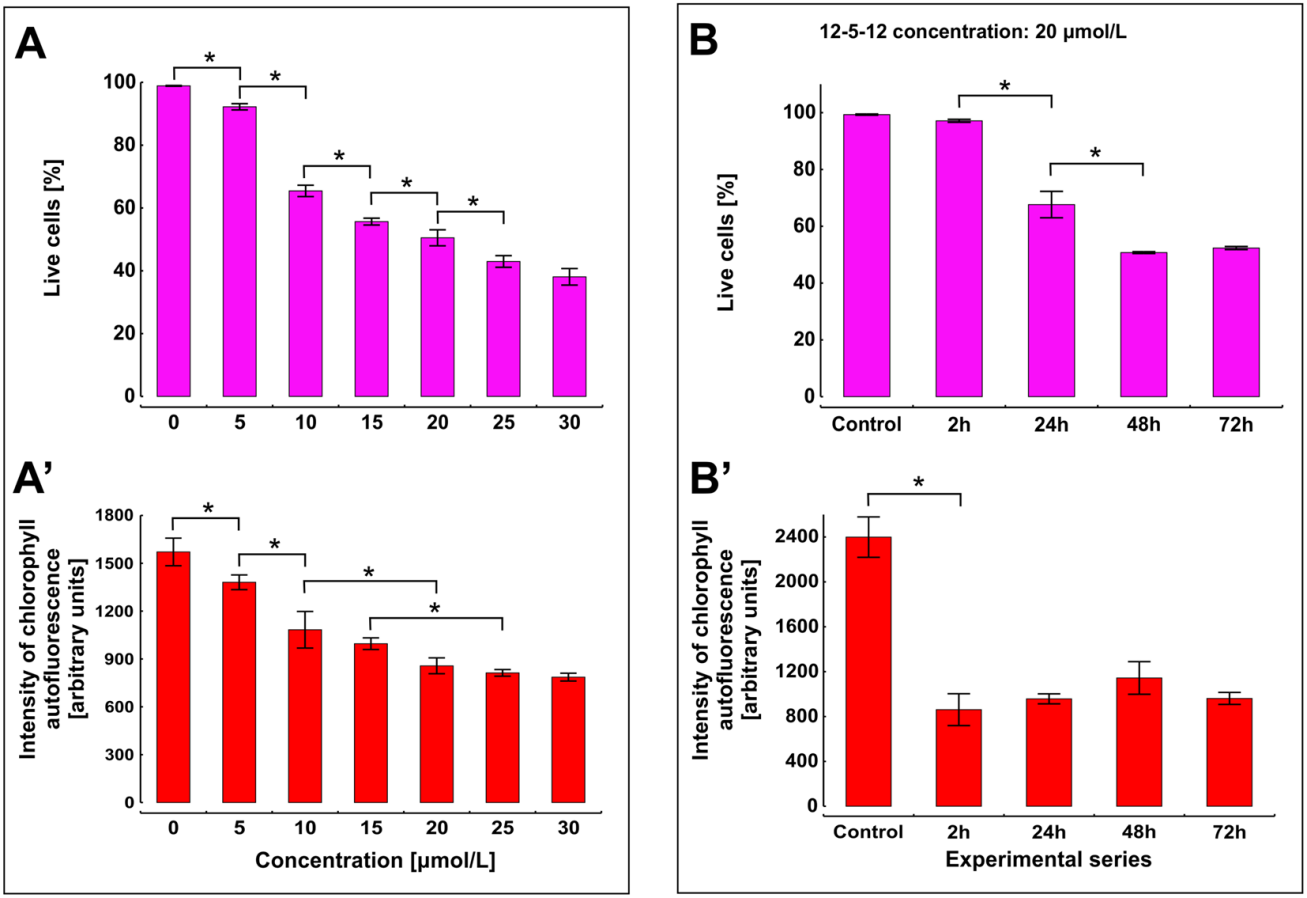
The changes in chlorophyll *a* autofluorescence after 72-h exposure to various concentrations of the 12-5-12 surfactant and during subsequent hours after exposure to the 12-5-12 surfactant at a concentration of 20 μmol/L. (**A**,**B**) The percentage of viable cells. (**A**’,**B**’) The intensity of chlorophyll *a* autofluorescence in the fraction of viable cells. The columns show mean values and the error bars represent standard deviation from three independent treatments. Statistical significance was assessed with the one-way ANOVA followed by the post-hoc Tukey's honestly significant difference (HSD) test. An asterisk indicates statistically significant differences (for **A**–**A**’ p < 0.05 and for **B**–**B**’ p < 0.01).

Not only the integrity of plasma membrane but the physiological condition of a photosynthetic apparatus was also investigated during the compound treatment. It is assumed that the reduction in chlorophyll *a* autofluorescence indicates damage of the photosynthetic apparatus^53^, thus it was monitored in a population of viable cells during exposure to the 12-5-12 surfactant. The obtained data indicate that in the fraction of viable cells (Fig. 6A) chlorophyll *a* autofluorescence declined with the increasing compound concentrations (Fig. 6A′). Furthermore, the highest decrease in this parameter (over 60%) was obtained already after 2-h exposure to the concentration of 20 µmol/L and it persisted during the following hours of treatment (Fig. 6B–B′). The obtained data seem to be in agreement with the previous research which indicated that detergents affect integrity of chloroplast membranes and reduce chlorophyll fluorescence in isolated photosystems^54–57^. Thus, the decrease in chlorophyll autofluorescence observed in our study right after the exposure to the 12-5-12 surfactant may indicate a dysfunction of the photosynthetic apparatus. However, one might ask why the 12-5-12 surfactant does not distinctly disrupt the plasma membrane but it affects mostly the functioning of chloroplasts?

Differences in lipid and fatty acid compositions of plasma membrane as well as of mitochondrial and chloroplast envelope could underlie various resistance reactions to the tested compound. Generally, detergent resistant membranes are rich in saturated fatty acids with a lower level of unsaturated ones^58,59^. Chloroplast lipids contain a relatively high percentage of polyunsaturated fatty acids in comparison to the other cell membranes. Furthermore, thylakoid membranes are rich in galactolipids, e.g. monogalactosyldiacyl-glycerol (MGDG) and digalactosyldiacylglycerol (DGDG). The former, due to its structure, does not favor the formation of a stable bilayer^60–62^. Thus, lipid composition could make chloroplast membranes more prone to detergent treatment.

It is possible that the 12-5-12 penetrates the plasma membrane without causing a serious damage of a lipid bilayer and the further loss of the plasma membrane properties results mostly from changes in cellular biochemistry triggered in response to reduced chloroplast functionality.

### The 12-5-12 surfactant affects the photosynthetic apparatus

To confirm that the 12-5-12 compound affects functioning of the photosynthetic apparatus we applied an indirect approach based on the nature of photochemical processes in chloroplasts. Energy harvested by photosystems may be emitted as chlorophyll fluorescence, however light energy exceeding photosynthetic capacity is dissipated into heat due to activation of fotoprotective mechanisms, e.g. the non-photochemical quenching (NPQ). Both the NPQ and the photoinhibition, which can be induced in extreme cases, were found to decrease efficiency of photoautotrophy^63–67^. Thus, if the exposure to the 12-5-12 surfactant triggers photosystems overexcitation and photoinhibition which are followed by decrease in cell viability, lower light intensity could diminish this effect and reduce the number of dead cells.

**Figure 7:**
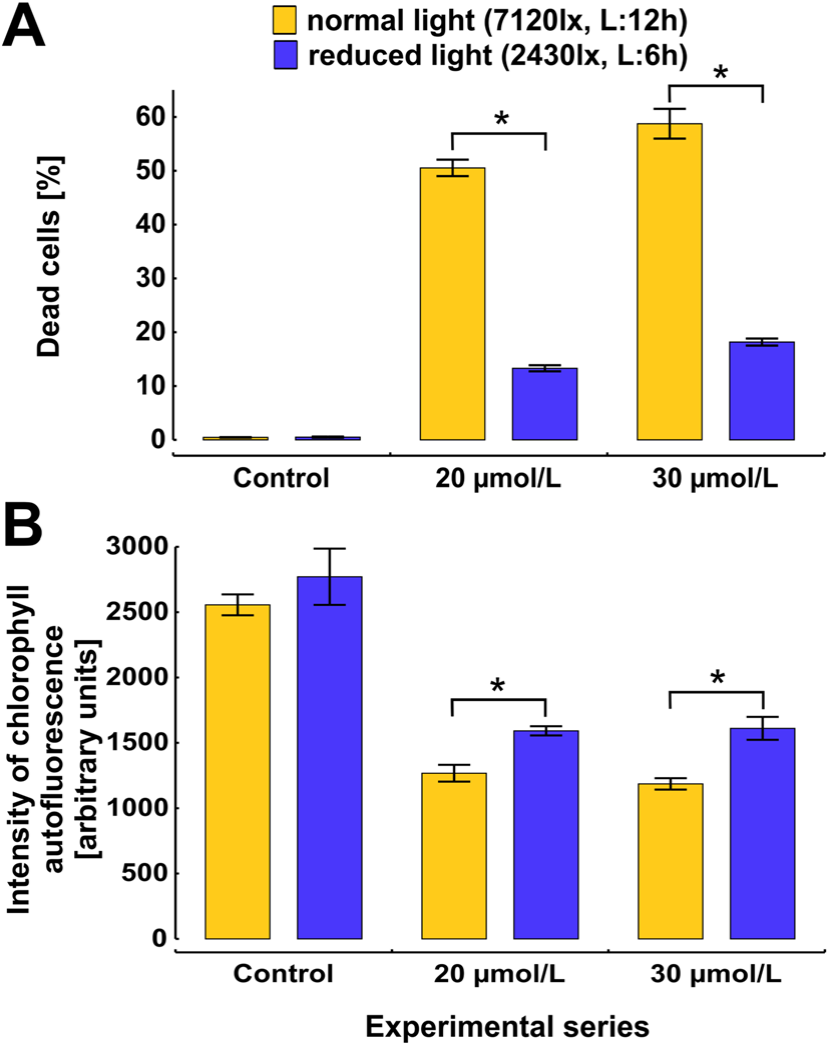
The effect of various light conditions on cell viability and chlorophyll *a* autofluorescence after 72-h exposition to different concentrations of the 12-5-12 surfactant. The percentage of dead cells (**A**) and the chlorophyll *a* autofluorescence in fraction of viable cells (**B**) at normal and reduced light conditions after exposure to the concentrations of 20 or 30 μmol/L of the 12-5-12 surfactant. The columns show mean values and the error bars represent standard deviation from three independent treatments. L refers to light, lx refers to lux. Statistical significance was assessed with the Student’s *t* test. An asterisk indicates statistically significant differences (p < 0.001 and p < 0.002 for (**A**) and (**B**) respectively).

**Figure 8:**
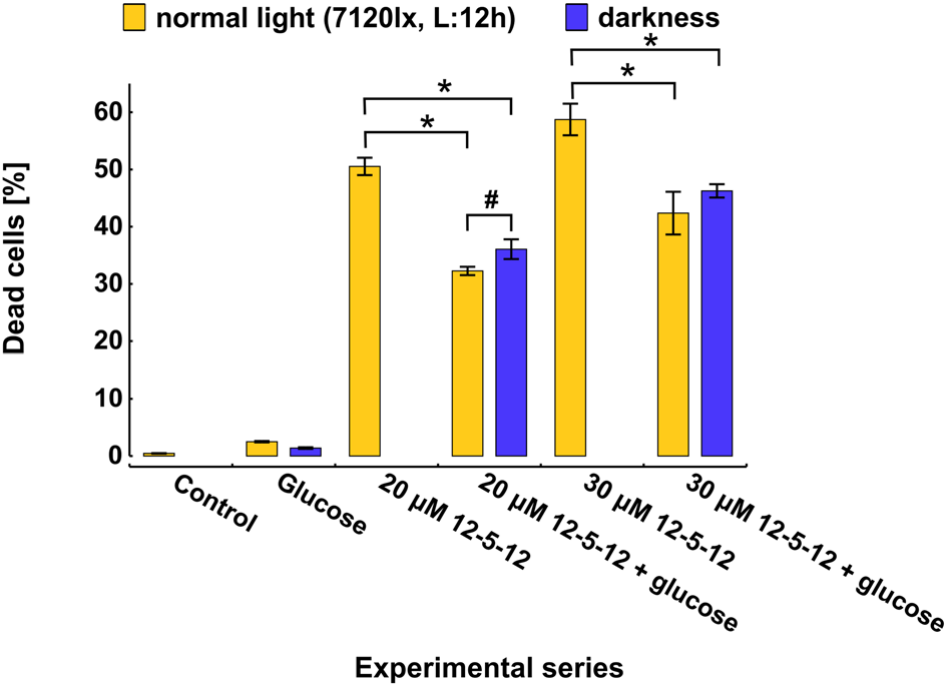
The effect of glucose and darkness on cell viability during 72-h exposition to various concentrations of the 12-5-12 surfactant. The columns show mean values and the error bars represent standard deviation from three independent treatments. L refers to light, lx refers to lux. Statistical significance was assessed with the Student’s *t* test. An asterisk and a number sign indicate statistically significant differences (p < 0.001 and p = 0.03 respectively).

The obtained data indicate that the percentage of dead cells decreased by about 70% at reduced light conditions during exposure to the concentration of 20 or 30 µmol/L (Fig. 7A). Interestingly, at the same time the intensity of chlorophyll *a* autofluorescence was slightly increased (Fig. 7B). Thus, the decrease in the number of dead cells at reduced light intensity may indicate that during exposure to the 12-5-12 surfactant, normal light conditions becomes the source of the high light stress which affects the functioning of photosynthetic apparatus and contributes to the following extensive cell death. Next, the algaecidal activity of the 12-5-12 surfactant was investigated towards cells which were grown in darkness. Unexpectedly, although dark conditions decreased the number of dead cells, the reduction was not as high as in the case of reduced light conditions. The fraction of non-viable cells declined from 50.6 to 36.1% and from 58.7 to 43.6% at the concentration of 20 and 30 µmol/L, respectively (Fig. 8). At the same time, the numbers of dead cells were also monitored at normal light conditions in the media supplemented simultaneously with the 12-5-12 surfactant and glucose to see whether it can reverse the negative impact of the reduced efficiency of photoautotrophy. The obtained data indicate that an addition of 1% glucose decreased the number of non-viable cells to the values similar to those observed at dark conditions. The fraction of dead cells declined to 32.3% and to 42.4% at the concentrations of 20 and 30 µmol/L, respectively. Interestingly, the cells treated with 20 μmol/L of the 12-5-12 surfactant showed a statistically significant difference between normal and dark conditions (Fig. 8).

The obtained data seems to be in agreement with the previous research which indicated that biosurfactants decrease the photosynthesis efficiency^68^. However, it is worth asking why darkness and glucose addition did not spectacularly reduce the number of dead cells as it was found at reduced light conditions. This observation could be explained by the fact that glucose added to media at light conditions was found to increase the level of MGDGs^69^, these of galactolipids which possibly make chloroplast more sensitive to detergents. Thus, although glucose addition diminishes the negative effect of sugar starvation caused by the 12-5-12 treatment, at the same time it can make chloroplast membranes less resistant to the surfactant. Furthermore in the presence of glucose a forward scatter (FSC) and a side scatter (SSC) signals indicate a reduction in cell size and an increase in cell granularity (Supplementary Fig. 1S), which may result from intensive cell divisions^70^ and the starch accumulation, respectively^71^. Interestingly, some contradictory studies indicate that the glucose addition to media may increase cell volume^71,72^. FSC and SSC signals must be interpreted with a caution, however it is still possible that autospores after hatching are less resistant to detergents due to a reduced thickness of cell wall or its specific chemical composition. Although cell wall thickness was slightly lowered in hatching autospores of Chlorella in comparison to mother cells^73^, more pronounced differences between mother and daughter cells were found in the case of *Parachlorella kessleri*^74^. Thus, intensive divisions in the presence of glucose might lead to a lower resistance of cell population to the 12-5-12 compound, which manifests itself in a higher than expected number of dead cells.

Another possible explanation may come from mitochondria which could be key players in this case. Although mitochondria contain more phospholipids and a lower percentage of highly unsaturated galactolipids^60,75^, which makes them more resistant to detergents than chloroplast, plant mitochondria were found to fuse and elongate in darkness^76^. In mammals and yeast cells mitochondria fusion seems to be crucial for maintaining their functionality in the case of high energy demand or under stress conditions^77,78^. The 12-5-12 surfactant might have a limited effect on respiratory capacity under light conditions, on the other hand it could impact mitochondrial envelope to the extent that may reduce their capability of fusion and functionality in darkness. However, this hypothesis is not directly supported by our results and could be investigated in the future.

Nevertheless, all of this indicates that the 12-5-12 surfactant affects functioning of the photosynthetic apparatus which seems to become sensitive to normal light conditions. Cell death observed in our study may result from sugar starvation, however changes in the overall cellular biochemistry, which could be the source of a secondary damage to plasma membrane, should be taken into consideration as well.

### The 12-5-12 induces reactive oxygen species (ROS)

**Figure 9:**
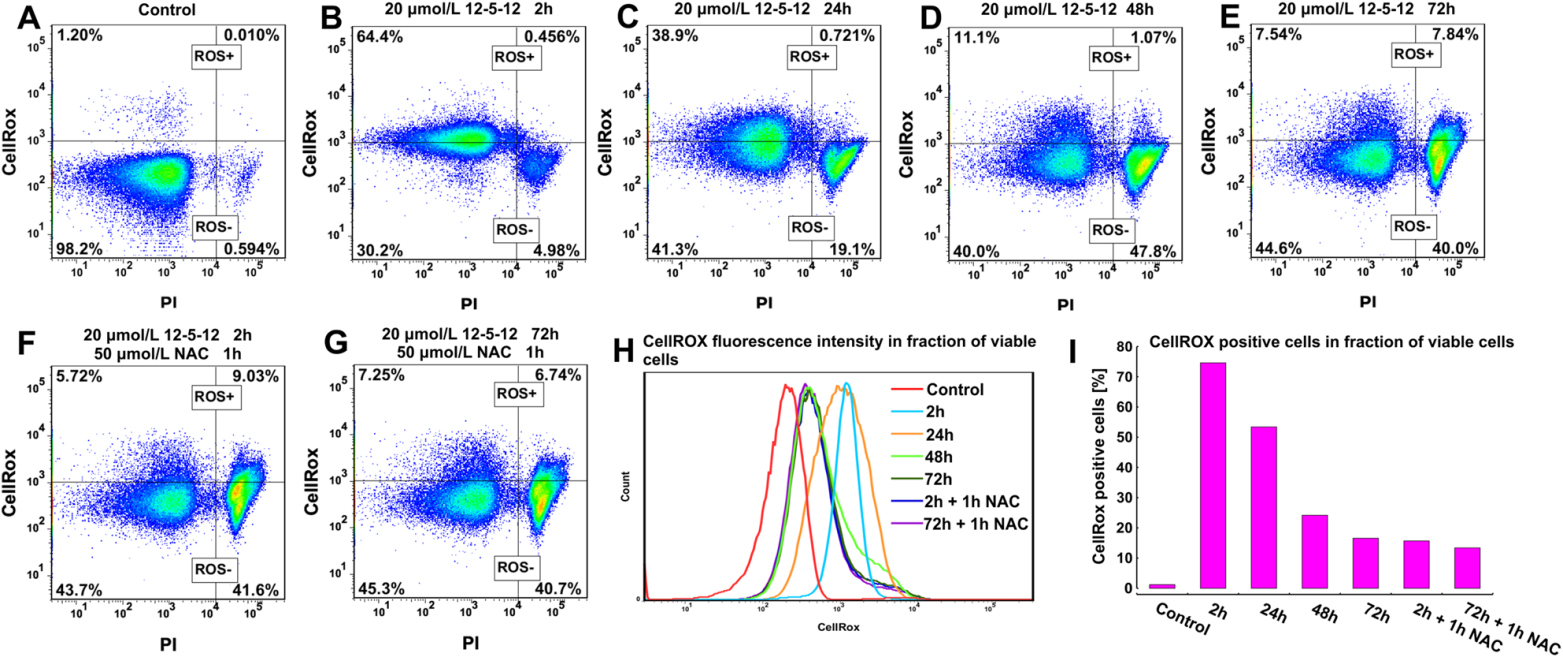
Reactive oxygen species (ROS) detection during subsequent hours of incubation with the 12-5-12 surfactant at the concentration of 20 μmol/L. (**A**–**E**) The diagrams show the percentage of ROS positive (+) and ROS negative (−) cells in the fractions of viable and non-viable cells after different times of exposure to the tested compound. (**F**,**G**) The diagrams show the percentage of ROS positive (+) and ROS negative (−) cells in the fractions of viable and non-viable cells after the exposure to the tested compound and treatment with 50 μmol/L NAC 1 h before analyses. (**H**) Changes in CellRox fluorescence intensity in the fraction of viable cells. (**I**) The percentage of CellRox positive cells in the fraction of viable cells. All diagrams show representative data obtained from two independent experiments.

Since damage of the photosynthetic apparatus may generate reactive oxygen species (ROS), their level was estimated during the exposure to the 12-5-12 surfactant at the concentration of 20 μmol/L. The obtained results indicate that an oxidative burst started already 2 h after the compound application and 76% of viable cells were gated as ROS positive (Fig. 9A,B). This effect was diminished when 50 µmol/L of the *N*-acetyl cysteine (NAC), an antioxidant, were added 1 h before ROS detection. Although NAC reduced ROS level, it simultaneously induced a considerable increase in the number of dead cells (Fig. 9B,F). During the following hours of exposure to the 12-5-12 surfactant, the percentage of viable cells with a high ROS content decreased to 16% after 72-h exposure (Fig. 9A–E,I). The reduction in the number of ROS positive cells was accompanied by a decrease in the fluorescence intensity. Although the number of ROS positive cells and the ROS level declined in the fraction of viable cells during the following hours of incubation, at the same time cell death increased distinctly (Fig. 9H). Interestingly, after 72-h treatment with the 12-5-12 surfactant, NAC added 1 h before ROS detection, was not able to reduce the number of ROS positive cells and fluorescence intensity (Fig. 9E,G,I).

**Figure 10:**
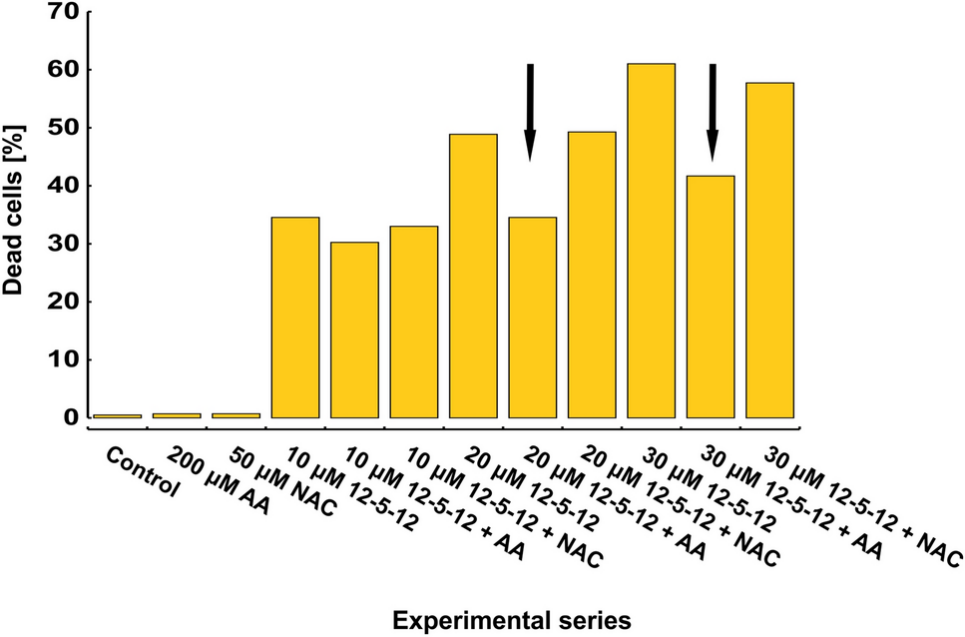
The effect of antioxidants on the number of dead cells. The percentage of dead cells was estimated after 72-h simultaneous exposition to various concentrations of the 12-5-12 and 50 μmol/L NAC or 200 μmol/L AA antioxidants. The columns represent data from a single experiment.

The observed alterations in the ROS level prompted us to investigate whether antioxidants present during the exposure to the 12-5-12 surfactant could reduce its algaecidal activity. Interestingly, 50 µmol/L NAC did not markedly decrease the number of dead cell, while 200 µmol/L ascorbic acid (AA) was found to reduce the fraction of non-viable cells to the levels similar to those observed after glucose addition at normal light conditions or in darkness. The impact of AA was the more visible the higher 12-5-12 concentration was used (Fig. 10). Thus, the obtained data clearly indicate that ROS participate in the 12-5-12-induced cell death. This finding is consistent with previous literature indicating that high light stress and following burst of ROS in chloroplasts may damage thylakoid lamellae and plasma membrane^79^.

In conclusion, the presented studies show the strong algaecidal activity of the 12-5-12 surfactant. The toxic effects were estimated on high cell density, indicating that the tested detergent could be a promising compound which reduces algal blooms at lower concentrations than other available agents. Our indirect approach, based on monitoring the number of dead cells at reduced light intensity, in darkness and after addition of glucose, may indicate that the 12-5-12 compound negatively affects functioning of chloroplast causing the reduction in efficiency of photoautotrophy and the increase in ROS level. Thus, the algaecidal activity of the 12-5-12 surfactant may result from two coexisting events: the reduction of photosynthesis intensity followed by a gradual deprivation of available sugar and ROS-induced secondary damages to plasma membrane. The former assumption seems to be supported by the fact, that sugar addition to the media decreased the number of dead cells. The latter may be underpinned by the fact that the burst of ROS appeared right after the compound application. However, further detailed analyses are needed to estimate which processes of photosynthesis are affected by the tested compound and close attention should be paid to the environmental impact of the 12-5-12 surfactant.

## Material and methods

### Chemical agents

The 12-5-12 compound was synthesized and prepared for biological investigations according to the previously described procedures^80^. Propidium iodide (PI), 3,3′-dihexyloxacarbo-cyanine iodide (DiOC6(3)), *N*-acetyl cysteine (NAC), l-ascorbic acid (AA) and glucose were obtained from Sigma. CellROX Green Reagent was derived from Thermo Fisher Scientific. Bold's Basal Medium (BBM) was supplied by Culture Collection of Autotrophic Organisms (CCALA) of the Institute of Botany of the Czech Academy of Sciences (AS CR). Other chemicals were obtained from POCH S.A.

### Algal strain and culture conditions

Chlorella vulgaris Beijerinck (896) was obtained from CCALA. The cells were propagated in 25 cm^2^ tissue culture flasks containing 8 mL BBM medium and illuminated with 7120 lx for 12 h/12 light/dark daily cycles. To obtain the desired density the cells were diluted in a fresh medium usually in the proportion 1:1. The cells treated with the 12-5-12 surfactant were grown on 12-well cell culture plates. During exposure the plates were shaken gently and illuminated with 7120 lx for 12 h/12 h light/dark daily cycles (denoted as normal light conditions) or with 2430 lx for 6 h/18 h light/dark daily cycles (denoted as reduced light conditions). The fitotron temperature was set to 21 °C.

### Estimation of cell density

Before each experiment, the initial cell density was estimated using Fuchs-Rosenthal Counting Chamber. Three independent countings were performed. Next, mean values of cell density and standard deviation were calculated.

### Cell viability

For each experimental series 1 mL of cell suspension was centrifuged (1500*g*, 10 min, 4 °C) and next the pellets were resuspended in 0.7 mL of PBS (pH 7.4). Then, the samples were stained with PI to a final concentration of 9 μmol/L for 30 min at room temperature in darkness. The cells were analysed with a flow cytometer (LSRII, Becton Dickinson, East Rutherford, NJ, USA). PI fluorescence was excited with a blue laser 488 nm and collected with a 610/20 emission filter in front of the detector. For each sample, 100,000 events were counted. The cells showing a high value of fluorescence were gated as non-viable ones. At the same time, chlorophyll *a* autofluorescence was excited with a violet laser 405 nm and detected with a 655/8 emission filter.

Cells treated with a very high concentration of surfactant were found to lose green pigment and these cells were used as a control to determine whether a laser 405 induces chlorophyll *a* autofluorescence. As indicated in supplementary materials, cells without chlorophyll which were excited with a laser 405 or 488 showed a low degree of the fluorescence intensity. In turn, cells containing chlorophyll displayed shift into a higher level of the fluorescence intensity after being excited with a laser 405 and detected with 655/8 emission filter. At the same time no increase in the fluorescence intensity was recorded after excitation with a laser 488 and detection with 610/20 emission filter. It may indicate that a detector with 610/20 emission filter does not collect a high percentage of the chlorophyll *b* fluorescence, which could be also excited with a laser 488. Only after the addition of PI, cells containing chlorophyll show a high level of the fluorescence intensity while excited with a laser 488 (Supplementary Fig. 2S). Furthermore, to check that cells counted as PI positive are not false positive due to chlorophyll *b* excitation, cells were also investigated under a fluorescence microscope. In these cells strong red fluorescence was localized in nuclei after surfactant treatment (Supplementary Fig. 3S).

The mean value of chlorophyll autofluorescence and the number of dead cells were estimated based on measurements of 100,000 cells. The data was exported and analyzed with FlowJo software.

### Measurements of plasma membrane potential

Plasma membrane potential was monitored with DiOC6(3) according to the previously described method^32^. Aliquots of the cell suspension were stained with DiOC6(3) to a final concentration 0.25 μmol/L for 1 h. To discriminate viable and non-viable cells, 9 μmol/L PI was added to the samples 30 min prior the flow cytometry analyses. Staining was performed at room temperature in darkness. PI fluorescence was measured as described above. DiOC6(3) fluorescence was excited with a blue laser 488 nm and gathered with a 530/30 emission filter. To correct the overlap between DiOC6(3) and PI fluorescence, a fluorescence compensation was performed. For each sample 100,000 events were counted. The data was exported and analyzed with FlowJo software. The cells showing a high value of fluorescence were classified as DiOC positive ones.

### Reactive oxygen species (ROS) detection

ROS were detected with the CellROX Green Reagent. The samples were incubated with the reagent at a final concentration of 5 μM for 1 h at 37 °C in darkness. Next, the cells were washed twice with PBS (pH 7.4) by centrifugation (1500*g*, 10 min, 4 °C). To discriminate viable and non-viable cells 9 μmol/L PI was added to the samples for 30 min prior the flow cytometry analyses at room temperature in darkness. PI fluorescence was measured as described above. CellROX Green Reagent fluorescence was excited with a blue laser 488 nm and gathered with the 530/30 emission filter. To correct the overlap between CellROX and PI fluorescence, a fluorescence compensation was performed. For each sample 100,000 events were counted. The data was exported and analyzed with FlowJo software. The cells showing a high value of fluorescence were gated as ROS positive ones.

### Statistical analyses

Statistical analyses were performed with Statistica 13 software. Normality was assessed with the Shapiro–Wilk test, and equality of variances was estimated with Brown–Forsythe test. Depending on a data set, the differences between mean values were assessed with the Student’s *t* test, the one-way or two-way analysis of variance (ANOVA) and followed by post-hoc Tukey’s test.

## Supplementary Information

Supplementary Figures.

## Data Availability

The datasets generated and analyzed during the current study are available from the corresponding author on reasonable request.
