# Supplementary material for: Anti-algal activity of the 12-5-12 gemini surfactant results from its impact on the photosynthetic apparatus
Source: Sci Rep. 2021 Jan 27;11:2360. doi: 10.1038/s41598-021-82165-9 (PMC7840743; doi:10.1038/s41598-021-82165-9)
Supplement: Supplementary file 1 — Supplementary Figures. [file 41598_2021_82165_MOESM1_ESM.pdf]

Anti-algal activity of the 12-5-12 gemini surfactant results from its impact on the photosynthetic apparatus

Konrad Krajewski [1,*], Katarzyna Łudzik [2,3], Aneta Żabka [1], Justyna Teresa Polit [1], Anna Zawisza [4], and Janusz Maszewski [1]

1. Department of Cytophysiology, Faculty of Biology and Environmental Protection, University of Lodz, ul. Pomorska 141/143, 90-236 Lódź, Poland
2. Department of Physical Chemistry, Faculty of Chemistry, University of Lodz, ul. Pomorska 163/165; 90-236 Łódź, Poland
3. Frank Laboratory of Neutron Physics, Joint Institute for Nuclear Research, Dubna, Russia
4. Department of Organic and Applied Chemistry, Faculty of Chemistry, University of Lodz, ul. Tamka 12, 91-403 Łódź, Poland

*Correspondence: e-mail: konrad.s.krajewski@gmail.com

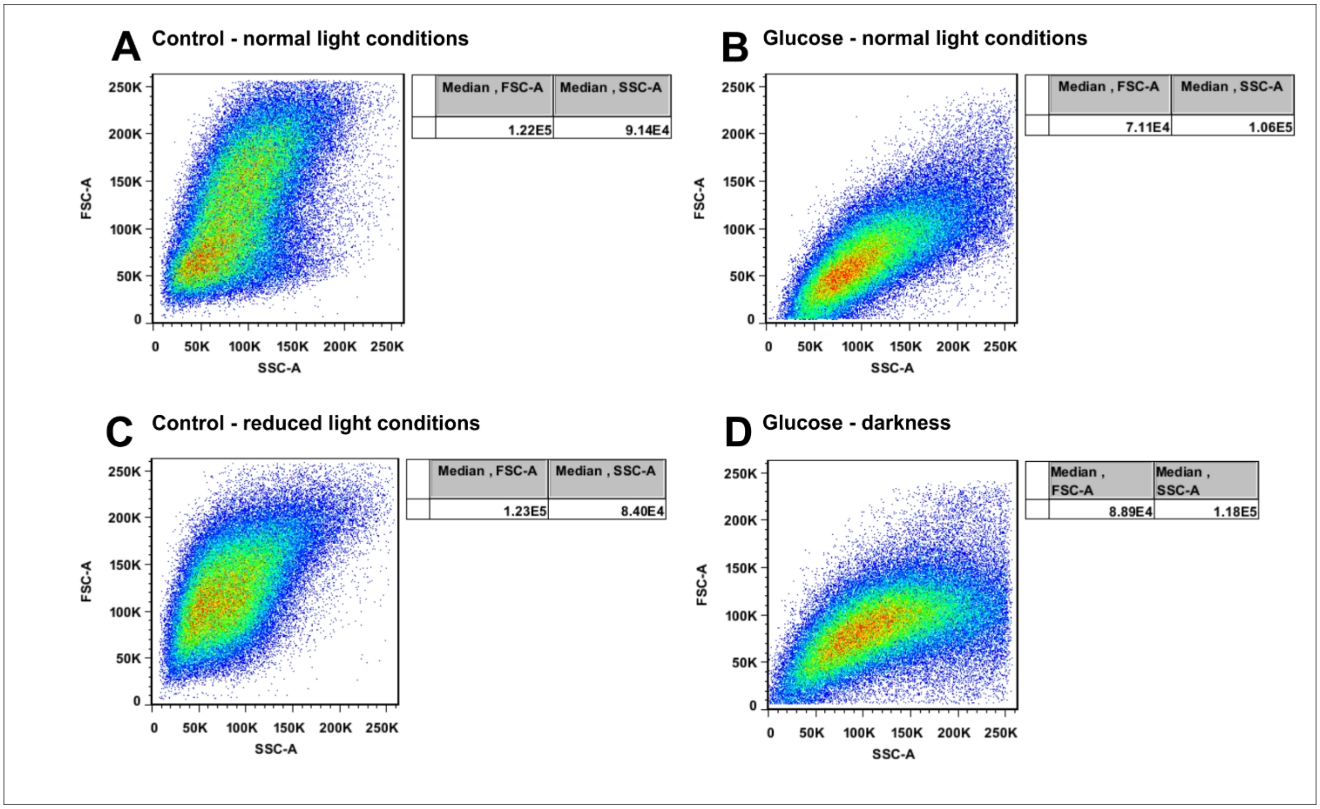

Fig. 1S FSC versus SSC plots showing a relative cell volume and granularity. (A) Cells grown at normal light conditions (7120lx, 12h/12h light/dark daily cycles). (B) Cells grown at normal light conditions in the presence of glucose. (C) Cells grown at reduced light conditions (2430lx, 6h/18h light/dark daily cycles). (D) Cells grown in darkness in the presence of glucose.

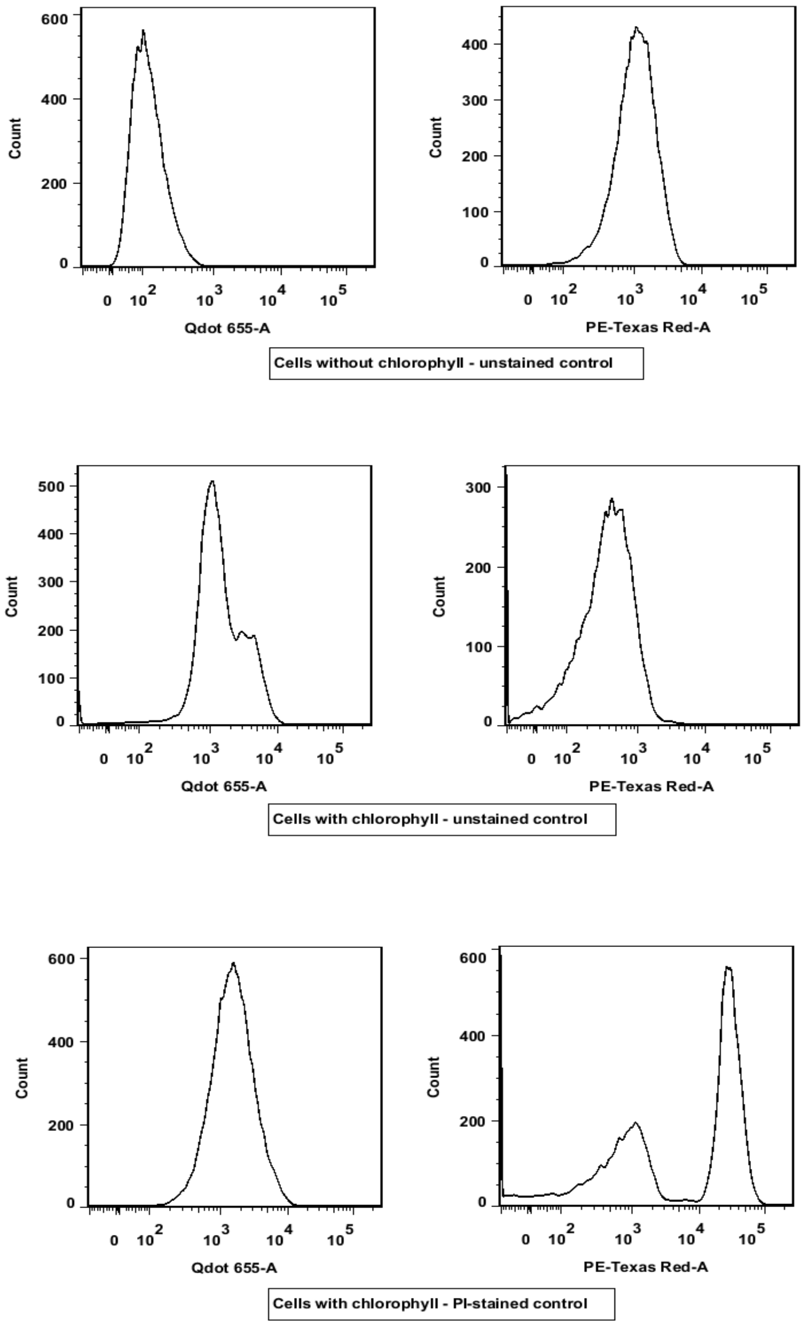

Fig. 2S Flow cytometry analyzes of the fluorescence intensity detected with a 655/8 emission filter denoted as Qdot 655 (for chlorophyll a) and a 610/20 emission filter denoted as PE-Texas-Red (for PI positive cells) after excitation with lasers 405 and 488, respectively. Cells without chlorophyll were obtained after incubation with the 12-5-12 at the concentration of 100 μmol/L (the upper panel). The middle panel presents non-treated cells (control). Dead cells in PI-stained control were obtained after incubation with the 12-5-12 at the concentration of 20 μmol/L (the bottom panel).

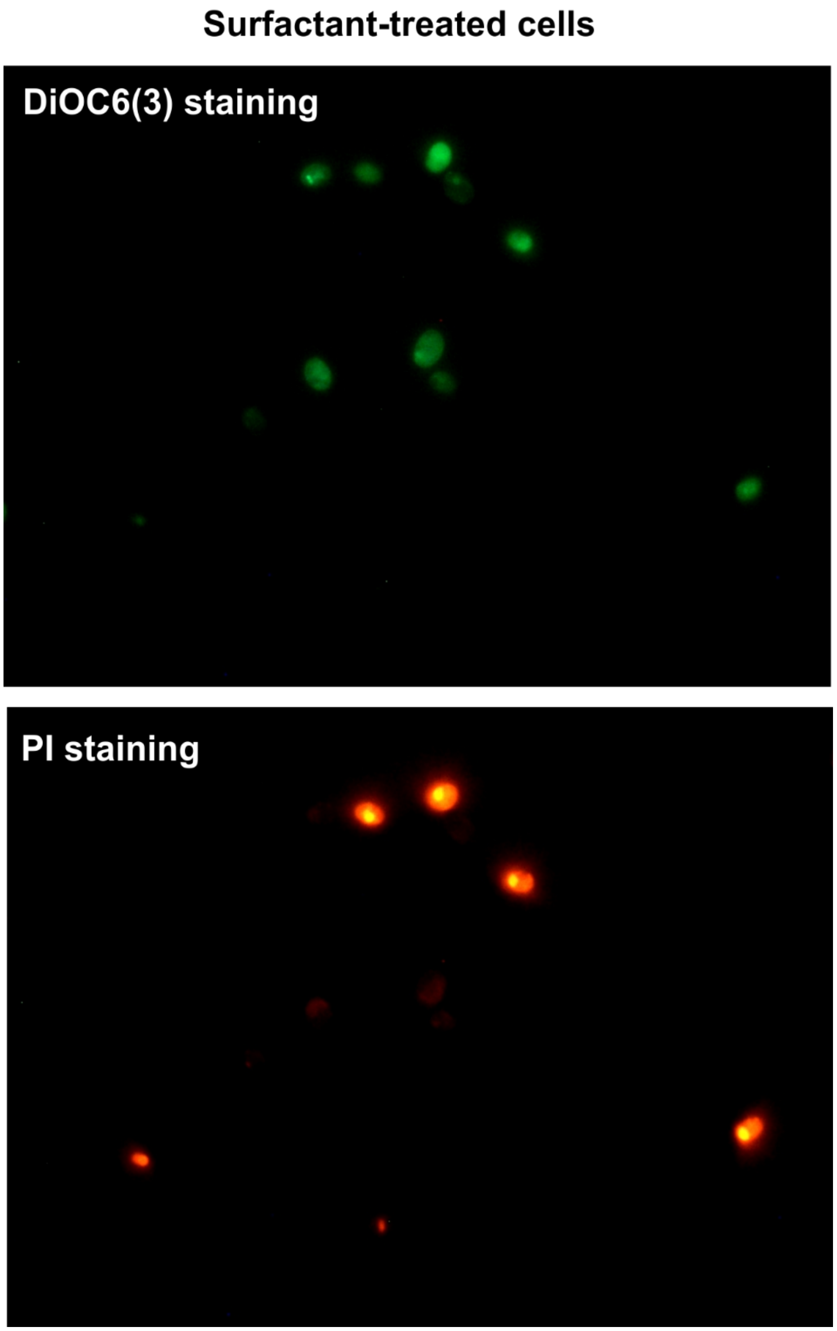

Fig. 3S

Green DiOC6(3) and red PI fluorescence in cells treated with 12-5-12 gemini surfactant observed under fluorescence microscopy. For DiOC6(3) fluorescence EX 465-495 and BA 515-555 filters were used and for PI florescence EX 540/25 and BA 605/55 filters were applied.
